# Magnesium alginate versus proton pump inhibitors for the treatment of laryngopharyngeal reflux: a non-inferiority randomized controlled trial

**DOI:** 10.1007/s00405-021-07219-0

**Published:** 2022-01-15

**Authors:** Nicole Pizzorni, Federico Ambrogi, Angelo Eplite, Sibora Rama, Carlo Robotti, Jerome Lechien, Antonio Schindler

**Affiliations:** 1 Phoniatric Unit, Department of Biomedical and Clinical Sciences “Luigi Sacco”, Università Degli Studi Di Milano, Via G.B Grassi, 74, 20157 Milan, Italy; 2 Laboratory of Medical Statistics, Biometry, Epidemiology “G.A. Maccararo”, Department of Clinical Sciences and Community Health, Università Degli Studi Di Milano, Via Vanzetti, 5, 20133 Milan, Italy; 3 Laboratory of Anatomy and Cell Biology, Faculty of Medicine, University of Mons (UMONS), Avenue du Champ de mars, 6, B-7000 Mons, Belgium

**Keywords:** Laryngopharyngeal reflux, Proton pump inhibitors, Alginates, Otolaryngology

## Abstract

**Purpose:**

Proton pump inhibitors (PPIs) are commonly prescribed for laryngopharyngeal reflux (LPR), but their efficacy remains debated. Alginates is an option for the treatment of LPR with few adverse effects. The study aimed to investigate the non-inferiority of an alginate suspension (*Gastrotuss*^®^) compared to PPIs (Omeprazole) in reducing LPR symptoms and signs.

**Methods:**

A non-inferiority randomized controlled trial was conducted. Fifty patients with laryngopharyngeal symptoms (Reflux Symptom Index -RSI- ≥ 13) and signs (Reflux Finding Score -RFS- ≥ 7) were randomized in two treatment groups: (A) *Gastrotuss*^*®*^ (20 ml, three daily doses) and, (B) Omeprazole (20 mg, once daily). The RSI and the RFS were assessed at baseline and after 2 months of treatment.

**Results:**

**Groups had similar** RSI and RFS scores at baseline. From pre- to 2-month posttreatment, the mean RSI significantly decreased (*p* = 0.001) in alginate and PPI group (*p* = 0.003). The difference between groups in the RSI change was not significant (95%CI:  − 4.2–6.7, *p* = 0.639). The mean RFS significantly decreased in alginate (*p* = 0.006) and PPI groups (*p* = 0.006). The difference between groups in the mean change RFS was not significant (95%CI:  − 0.8; 1.4, *p* = 0.608).

**Conclusion:**

After 2 months of treatment, LPR symptoms and signs are significantly reduced irrespective of the treatment. A*lginate* was non-inferior to PPIs and may represent an alternative treatment to PPIs for the treatment of LPR.

## Introduction

Laryngopharyngeal reflux (LPR) is an inflammatory condition of the upper aerodigestive tract tissues caused by the direct and indirect effects of gastroduodenal content reflux, which may induce morphologic changes in the interested tract [1]. Common laryngeal findings are arytenoid and vocal cord erythema, posterior commissure hypertrophy, and arytenoid oedema [2–4]. Patients with LPR often experience hoarseness, globus sensation, throat clearing, cough, excess throat mucus, and postnasal drip [4]. LPR is associated with a poor quality of life and a significant healthcare cost [1, 4]. Although the prevalence of LPR is still unclear due to a lack of a gold standard for its diagnosis, it was estimated that LPR represents up to 10% of otorhinolaryngologists’ consultations [6, 7]. Additionally, an increase in the number of medical visits because of reflux, either LPR or gastroesophageal reflux disease (GERD), and in the number of anti-reflux prescriptions has been observed over the last decades [8], suggesting that reflux is an increasingly spread health issue.

Treatment options include diet, behavioural modifications and medication. Currently, proton pump inhibitors (PPIs) are the most commonly prescribed pharmacological treatment [9]. Nevertheless, the efficacy of PPIs in the treatment of LPR is still a matter of debate. In 2016, the meta-analysis by Liu and colleagues based on 8 randomized controlled trials (RCTs) concluded that PPIs did not significantly reduce LPR symptoms compared to the placebo [10]. The meta-analysis by Wei pooled the results of 13 RCTs and demonstrated a superiority of the PPIs over placebo for the treatment of LPR symptoms, as measured by the Reflux Symptom Index (RSI) [11], but a non-significant difference between the two groups for the response rate [12]. More recently, a meta-analysis by Lechien and colleagues, including 10 RCTs, supported a mild superiority of PPIs over placebo for the treatment of LPR, but also suggested that a higher response rate can be achieved by combining PPIs and behavioural modifications [1]. Moreover, the long-term use of PPIs is suspected to be associated with side effects, e.g. pneumonia, *Clostridium difficile* infection, kidney disease, heart failure, micronutrient deficiency, neurological disorders, and low bone mineral density [13–15]. Consequently, other pharmacological approaches have been explored, including alginate, magaldrate, histamine-2 receptor antagonists, and prokinetics. Emerging literature suggests an emerging role of alginates for the treatment of LPR. Studies have investigated their efficacy against placebo [16, 17] or their use alone against the combination on PPIs and alginates [18]. However, to the best of our knowledge, no study compared the efficacy of alginates to the efficacy of PPIs in patients with LPR.

The aim of this study was to compare the efficacy of alginate suspension (*Gastrotuss*^®^) versus PPI (Omeprazole) in reducing symptoms and signs of LPR after 2-month treatment. Secondary aims were to compare the tolerability and the adherence to the two treatments. It was hypothesized that the efficacy of treatment of LPR symptoms and signs using *Gastrotuss*^®^ would be non-inferior to the use of PPI and that both treatments would be well tolerated and would exhibit satisfactory adherence.

## Material and methods

A non-inferiority RCT with two parallel arms (1:1) and single blinding (assessor) was conducted on consecutive adults with LPR and symptoms. The study was carried out according to the Declaration of Helsinki and was previously approved by the Institutional Review Board of the hospital (n.2017/ST/068). All participants provided written informed consent. The randomized controlled trial is reported according to the CONSORT statement [19].

### Patients

Patients were consecutively recruited from an Ear, Nose and Throat (ENT) ambulatory of a University hospital. The following inclusion criteria were considered: adults aged 18–70 years, symptoms and signs of LPR based on a RSI ≥ 13 [11, 20] and a Reflux Finding Score (RFS) ≥ 7 [21], with at least 15-day wash-out from other LPR treatments (i.e. PPI, prokinetic agents, histamine H2 receptor agonist, etc.). Exclusion criteria consisted of anti-reflux surgery, esophageal surgical procedure, major laryngeal/pharyngeal surgery, cancer, immunosuppression, immunodeficiency, diabetes, cystic fibrosis, vocal alterations not related to LPR, any concurrent cardiac, gastroenterological, respiratory, laryngeal, and oropharyngeal disease, drug and alcohol abuse, pregnancy, and breastfeeding.

### Treatments

Patients in the experimental group were treated with *Gastrotuss*^®^ (Drugs Mineral and Generics, Pomezia, Rome, Italy) 20 ml, three doses a day, after meals. *Gastrotuss*^®^ is a liquid preparation for oral administration, registered as a medical device, composed of an association of different agents including magnesium alginate and simethicone.

Patients in the control group were treated with PPI, Omeprazole 20 mg, a morning single daily dose in fasting condition.

Patients from both groups were provided with diet and lifestyle recommendations, according to the guidelines of the American College of Gastroenterology [22] (Appendix).

### Outcomes

Symptoms as measured by the RSI represented the primary outcome of the study. Secondary outcomes were videolaryngoscopic findings of LPR as measured by the RFS, the treatment’s tolerability, and the patients’ adherence to the treatment.

#### Reflux symptom index (RSI)

The RSI [11, 20] is a self-administered outcome measure developed to investigate LPR symptoms and their response to therapy. The RSI investigates the following symptoms: (1) hoarseness or voice problem; (2) throat clearing; (3) excess throat mucus or postnasal drip; (4) difficulty swallowing food, liquids or pills; (5) coughing after eating or lying down; (6) breathing difficulties or choking episodes; (7) troublesome or annoying cough; (8) sensation of something sticking or a lump in the throat; (9) heartburn, chest pain, indigestion or stomach acid coming up. Each symptom is rated on a six-point Likert scale ranging from 0 (no problem) to 5 (severe problem), with a total score ranging from 0–45. A total RSI score ≥ 13 is considered to be suggestive for LPR.

#### Reflux finding score (RFS)

The RFS [21] is an 8-item clinical severity scale developed to document the physical findings of LPR based on the fiberoptic laryngoscopy and their severity. The items assess: (1) subglottic oedema; (2) ventricular obliteration; (3) erythema/hyperemia; (4) vocal fold oedema; (5) diffuse laryngeal oedema; (6) posterior commissure hypertrophy; (7) granuloma/granulation tissue; (8) excessive endolaryngeal mucus. The scale ranges from 0 (no abnormal findings) to a maximum of 26 (worst score possible). A total RFS score ≥ 7 is considered to be suggestive for LPR. RFS was rated t the end of the study by two independent ENTs blinded to the patients’ allocation and the stage of the study.

#### Tolerability and Adherence

Tolerability was defined as “the degree to which overt adverse effects can be tolerated by the subject” [23]. Adherence to the pharmacological treatment was defined as “the extent to which a person's behaviour — taking medication, following a diet, and/or executing lifestyle changes — corresponds with the agreed recommendations from a healthcare provider” [24]. Tolerability and adherence were measured by a visual analogue scale (VAS) with 10 representing a greater tolerability of or adherence to the treatment. Moreover, the number of patient-reported adverse events were investigated. Adverse events were defined as any unfavourable and unintended sign, symptom, or disease temporarily associated with the use of a drug, without any judgement about causality or relationship to the drug.

### Study design and protocol

Patients accessing the ENT ambulatory clinic with symptoms of LPR were asked to complete the RSI and a fiberoptic laryngoscopy was performed to identify laryngeal findings of LPR scored according to the RFS (Month 0). Laryngoscopies were recorded for subsequent analysis. In case both the RSI and the RFS were positive for LPR, patients would be invited to participate to the study and to provide written informed consent. Data on age, gender, height and weight for body mass index (BMI) calculation, previous treatments for LPR, and concomitant gastroesophageal reflux diseases were recorded. Recruited patients were randomly allocated in a 1:1 ratio (simple randomization) to either *Gastrotuss*® or PPI by means of numbered, opaque, sealed envelopes. Diet and lifestyle recommendations were presented to each patient by the ENT who performed the laryngoscopy and a written report of the recommendations was provided. Patients were re-assessed after the 2 months (Month2, 58 days ± 2 days) of treatment. The Month2 assessment comprised the assessment of LPR symptoms through the RSI and a video-recorded fiberoptic laryngoscopy for subsequent RFS scoring. BMI was monitored. Patients were asked to rate on a VAS the tolerability and the adherence to the treatment and to report if they experienced adverse events.

### Statistical analysis

The quadratic weighted kappa was used to assess RFS inter-rater reliability. Baseline characteristics of the two groups of treatment were compared using Mann–Whitney U test for continuous variables and Chi-squared test for categorical variables. Paired parametric and non-parametric tests were used to test the significance of the 2 months change in RSI and RFS from baseline in each of the two trial arms. Analysis of covariance (ANCOVA) was used to evaluate the difference in the score change at 2 months follow-up between the two arms after adjusting for the baseline score. The test for difference in slopes for baseline measurements between treatment groups was evaluated through an interaction between baseline and trial arm, the normality of residuals with the Shapiro–Wilk test, and the homogeneity of variances with Levene test. The chi-squared test was used to compare the number of patients with an RSI score < 13 and an RFS score < 7 after LPR treatment. Tolerability and adherence VAS ratings were compared between the two trial arms using Mann–Whitney U test. Significance was set at *p* < 0.05.

### Sample Size

In the trial by Mcglashan et al*.* [16] a difference in reduction in RSI of 7 (with standard deviation 9) versus placebo was used as a clinically relevant difference. The non-inferiority threshold was set at 4 in this study, considering a margin almost halved compared to that of superiority. With a significance level of 5% and a power of 80%, a sample of 64 patients per group was planned to evaluate the non-inferiority of *Gastrotuss*^®^ compared to PPI. The surge of the pandemic of SARS-COV-2 did not allow to continue the trial that was stopped after the recruitment of 25 patients per group with complete follow-up at two months for 18 subjects in the *Gastrotuss*^*®*^ group and 22 patients in the PPI group. Such a sample size achieves about 40% power for the planned analysis while the 80% power is obtained with a larger non-inferiority margin equal to 7.

## Results

### Patients

**Fig. 1:**
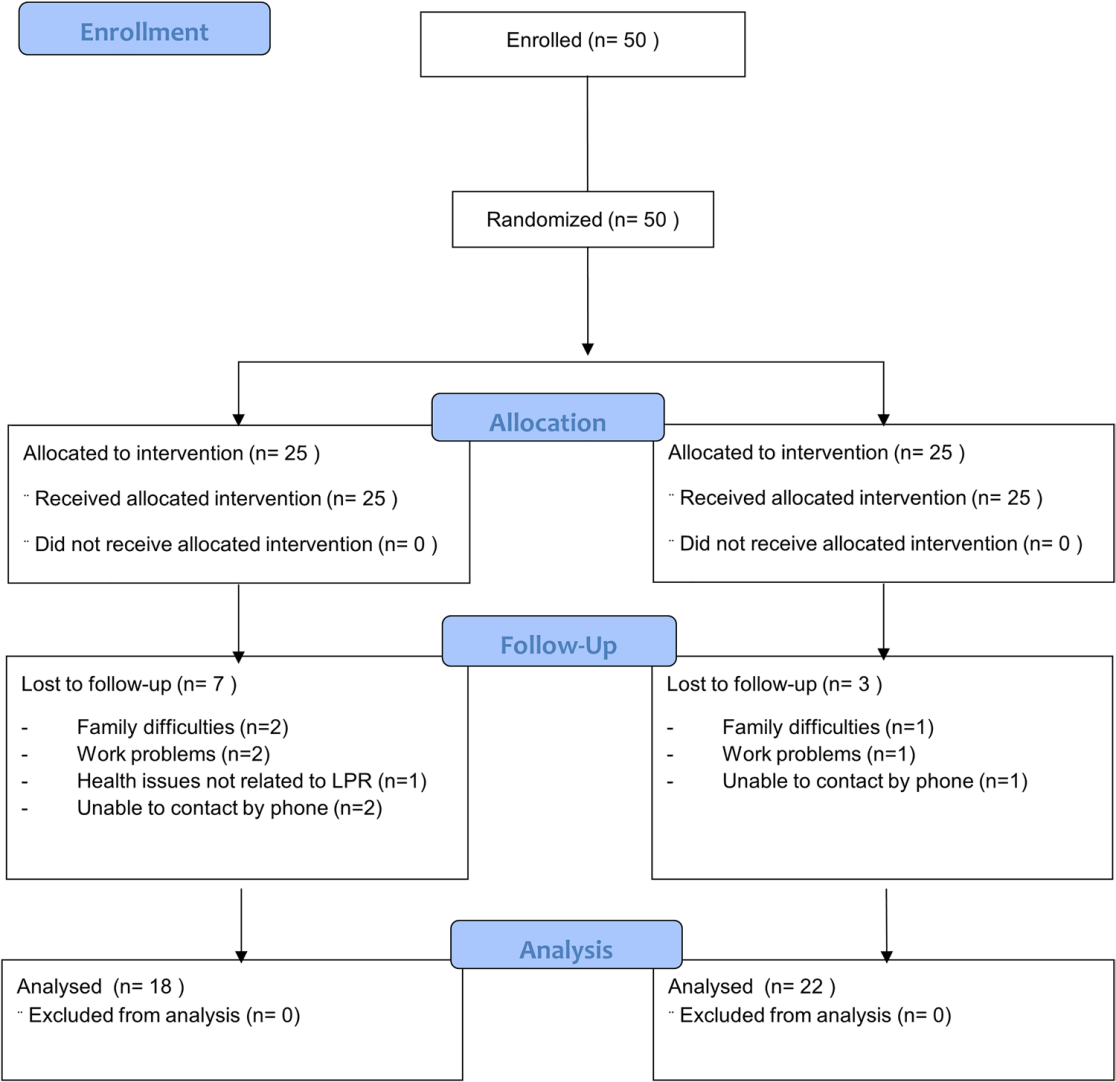
Patient’s sample flow-chart

Fifty patients (25 in the *Gastrotuss®* group and 25 in the PPI group) were recruited from July 2018 to February 2020. Afterward, recruitment was stopped because of the pandemic of SARS-COV-2. The flow-chart describes the patients’ sample in the different phases of the trial (Fig. 1). Age and BMI were similar in the two treatment groups while gender, alcohol and tobacco consumption were slightly unbalanced (Table 1). At the 2-month assessment, BMI variation was -0.02 (IQR -0.3—+ 0.03) in the *Gastrotuss®* group and -0.01 (IQR-0.04—+ 0.05) in the PPI group (*p* = 0.271).

**Table 1:** Baseline characteristics in the two treatment groups

	*Gastrotuss*^®^ (*n* = 25)	PPI (*n* = 25)	*p*
Age	68.5 (53.5–78.8)	70 (60–85)	0.123
Gender (M)	6 (24%)	9 (36%)	0.355
BMI	24.8 (22–26.7)	25.6 (22.9–28.6)	0.250
Alcohol consumption	4 (16%)	9 (36%)	0.186
Tobacco consumption	3 (12%)	6 (24%)	0.350

Legend. PPI, proton pump inhibitors; M, male; BMI, body mass index

### Primary outcome: RSI change

**Fig. 2:**
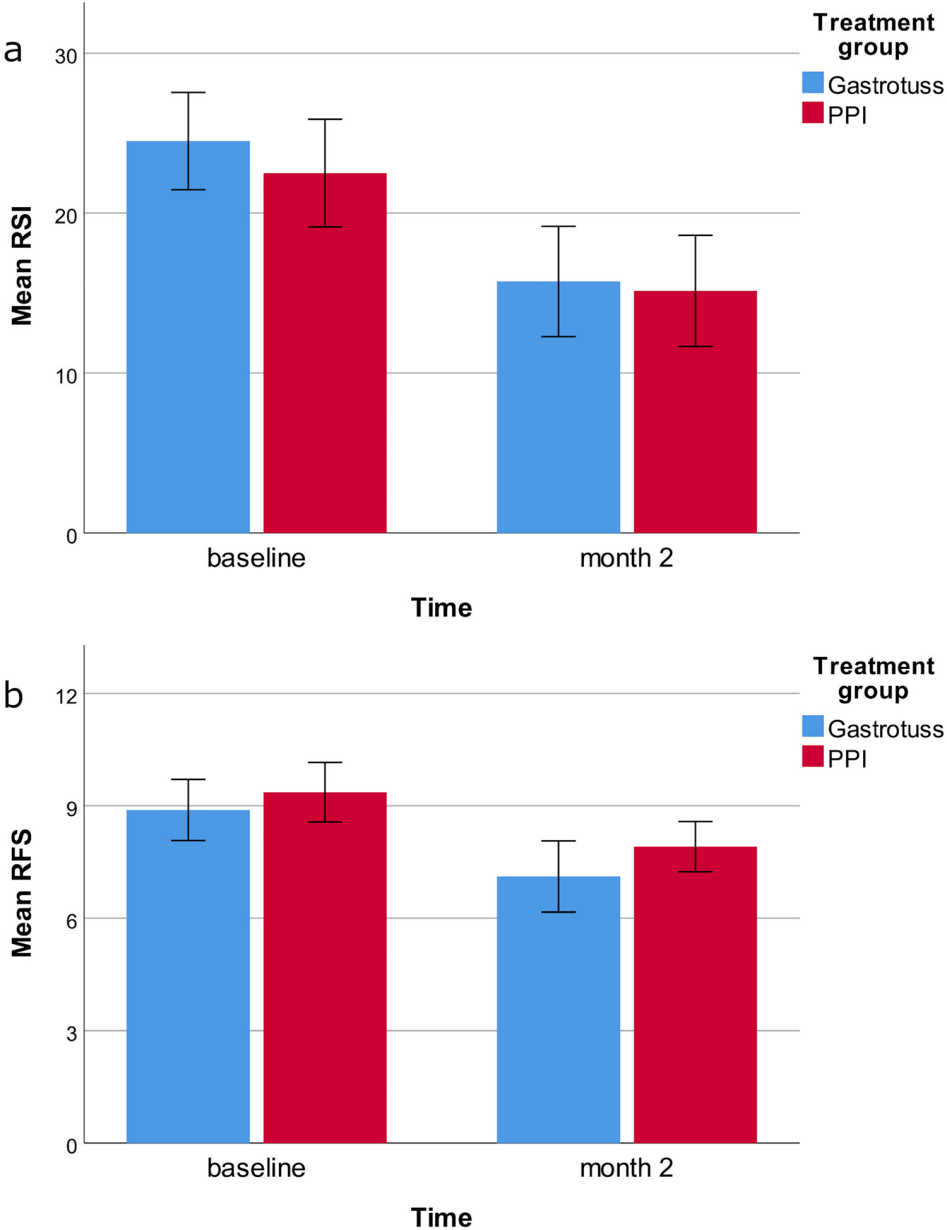
Reduction in RSI and RFS from Month 0 to Month 2 (ITT population). A. Change in the RSI from baseline to month 2 in the *Gastrotuss*^*®*^ group (blue; *n* = 18) and the PPI (red; *n* = 22) group. Bar errors represent the 95%CI. B. Change in the RFS from baseline to month 2 in the *Gastrotuss*^®^ group (blue; *n* = 18) and the PPI (red; *n* = 22) group. Bar errors represent the 95%CI.

The change in RSI from Month 0 to Month 2 is summarized in Fig. 2.

The RSI at baseline was similar in the two randomized groups. At Month 2, the mean RSI for *Gastrotuss*^*®*^ treated patients decreased by 8.5 ± 6.5 points (baseline 24.6 ± 6.1; month 2 16.1 ± 7.2; Wilcoxon exact paired test *p* = 0.001). For the PPI treated patients, mean RSI at Month 2 had decreased by 7.2 ± 9.8 points (baseline 22.5 ± 7.8; month 2 15.3 ± 8.2; Wilcoxon exact paired test p = 0.003).

The difference between the treatment groups in the mean change RSI from baseline to month 2 was 1.3 (95%CI: -4.2; 6.7, *p* = 0.639).

At month 2, an RSI score < 13 was reported by 6/18 (33.3%) patients in the *Gastrotuss*^*®*^ group and by 7/22 (31.8%) patients in the PPI group (*p* = 0.919).

According to ANCOVA (with baseline RSI as a covariate), the difference in mean change from baseline (*Gastrotuss*^*®*^—PPI) was -0.04 (95%CI  − 4.8; 4.8). Considered the original threshold, non-inferiority is not shown by a little amount while it can be considered achieved according to the recalculated threshold equal to 7.

**Fig. 3:**
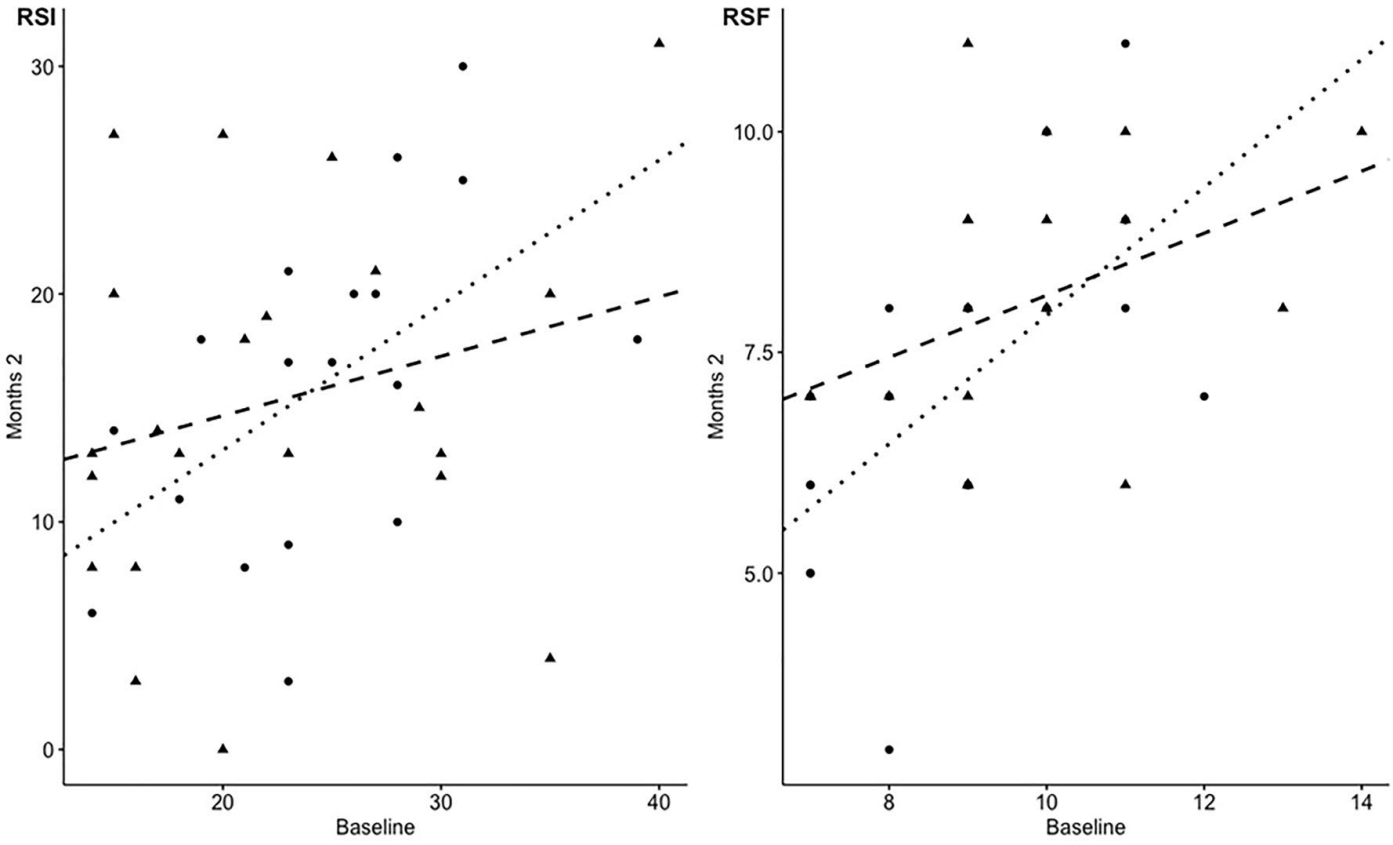
Scatter plot showing baseline and 2 months RSI (right panel) and RFS (left panel) scores for patients in *Gastrotuss*^®^ (bullets) and PPI (triangles) arms. Two regression lines are shown, the dotted one for *Gastrotuss*^®^ and the dashed one for PPI. The difference between the slopes of the two lines is not significant (RSI: *p* = 0.301; RFS: *p* = 0.185)

Regarding ANCOVA, no evidence of difference in slopes for baseline measurements between treatment groups (*p* = 0.301), of non-normality of residuals (*p* = 0.975) and non-homogeneity of variances (*p* = 0.504) were shown (Fig. 3).

### Secondary outcome: RFS change

Inter-rater reliability for the total RFS was k = 0.794 ± 0.038. The change in RFS from Month 0 to Month 2 is summarized in Fig. 2. The RFS at baseline was similar in the two randomized groups. At Month 2, the mean RFS for *Gastrotuss®* treated patients decreased by 1.8 ± 1.6 points (baseline 8.9 ± 1.6; month 2 7.1 ± 1.9; Wilcoxon exact paired test *p* = 0.006). For the PPI treated patients, mean RFS at Month 2 had decreased by 1.5 ± 1.8 points (baseline 9.5 ± 1.8; month 2 8.0 ± 1.5; Wilcoxon exact paired test *p* = 0.006). The difference between the treatment groups in the mean change RFS from baseline to month 2 was 0.3 (95%CI: -0.8; 1.4, *p* = 0.608). At month 2, an RSI score < 7 was reported by 7/18 (38.9%) patients in the *Gastrotuss*^*®*^ group and by 4/22 (18.2%) patients in the PPI group (*p* = 0.145). According to ANCOVA (with baseline RFS as a covariate), the difference in mean change from baseline (*Gastrotuss*^*®*^—PPI) was -0.56 (95%CI-0.4; 1.5). Regarding ANCOVA assumptions, no evidence of difference in slopes for baseline measurements between treatment groups (*p* = 0.185), of non-normality of residuals (*p* = 0.585) and non-homogeneity of variances (*p* = 0.590) were shown (Fig. 3).

### Tolerability and Adherence

No significant differences were detected between the two treatment groups regarding tolerability and adherence. Median tolerability was 8.5 (IQR 8–9.3) in the *Gastrotuss®* groups and 9 (IQR 8–9.3) in the PPI group (p = 0.583). Only 2 mild adverse events were reported for the *Gastrotuss®* group (nausea, heartburn). Median adherence was 8 (IQR 8–9) in the *Gastrotuss®* group and 8 (IQR 8–9) in the PPI group (p = 0.379).

## Discussion

PPIs are the most commonly prescribed treatments for LPR [9], but the PPI efficacy remains debated and long-term use has been associated with potential adverse events [9, 10, 12–15]. Consequently, investigation on alternative treatments is a current challenge. Recently, alginates emerged as an option for the treatment of LPR [16, 18]. For the first time, the present non-inferiority RCT compared the efficacy of PPIs (omeprazole) and the efficacy of the alginates suspension *Gastrotuss®* for the treatment of symptoms and signs of LPR. Symptoms, as measured by RSI, and signs, as measured by RFS by a blinded rater, were significantly reduced after 2 months of pharmacological treatment associated with diet and lifestyle recommendations compared to the baseline in both groups. The mean change in the RSI and the RFS were similar in the two treatment groups. Tolerability and adherence were high, with comparable ratings between PPIs and *Gastrotuss*^*®*^.

Both the *Gastrotuss*^®^ and the omeprazole significantly reduced the RSI score, which was normalized in around one-third of each treatment group. This finding confirmed the results of other studies using similar end-points. Overall, literature shows a good response of symptoms to LPR treatments. Concerning alginates, McGlashan et al*.* conducted a RCT on 49 patients comparing the efficacy of a liquid alginate suspension (*Gaviscon® Advance*) to placebo in reducing LPR signs and symptoms [16]. They found a superiority of the alginate for LPR symptoms as measured by the RSI both at 2 and 6 months. A significant reduction of the RSI was reported also by Tseng et al*.* in a RCT comparing alginates (*Alginos*) to placebo after 8-weeks of treatments in 80 patients with LPR, although it did not significantly differ to the RSI reduction of the placebo [17]. Another study compared the effect of the alginate (*Gaviscon*^*®*^* Advance*) alone to the efficacy of the alginate as an add-on treatment to PPIs in 72 patients with LPR [18]. After 3 months, the authors observed a reduction of LPR symptoms, as measured by the RSI, in around 90% of the sample with no significant difference between the treatment groups. With regards to PPIs, the RSI score was significantly reduced in the study by Reichel and colleagues comparing the efficacy of 3-months treatment with esomeprazole to placebo [25]. Other studies on PPIs using non validated tools reported a significant improvement of laryngeal and pharyngeal symptoms after two months of either omeprazole [26] or rabeprazole [27] and after 3 months of pantoprazole [28, 29].

Literature suggests that videolaryngoscopic findings of LPR require longer treatment period than subjective symptoms to show a treatment response [30]. Nevertheless, a significant reduction of laryngeal signs of LPR was observed after two months of treatment with both the alginate and the PPI, with a mean change of the RFS of 1.8 points and 1.5 points, respectively. A similar decline of the RFS score was reported in the study by MacGlashan et al*.* [16] and by Tseng et al*.* [17] following two months of alginates. Interestingly, concerning the PPI, this result was achieved with a low dose of omeprazole (20 mg, once a day). Other studies using higher doses of PPIs failed to detect a significant improvement in videolaryngoscopic findings after the same treatment period. In the trial by Noordzij and colleagues assessing the efficacy of 40 mg omeprazole twice a day, none of the laryngeal signs of LPR significantly changed over the course of the study [26]. However, the baseline laryngoscopic assessment already showed mild objective laryngeal inflammation. The RCT by Wo and colleagues analysed the efficacy of a 12-week treatment with 40 mg pantoprazole and reported no change in the RFS score in the treatment groups, despite the patients reported a relief of LPR symptoms [28]. Steward and colleagues conducted a RCT comparing 20 mg rabeprazole twice a day to placebo [27]. They reported an improvement of laryngeal signs, but it did not reach the statistical significance. These differences may be related to the severity of laryngeal findings or to the different assessment scales used. Another hypothesis is that the greater response of the present study may be attributable to the patients’ selection. Indeed, inclusion criteria for the study were very restrictive and patients with concomitant diseases that may represent confounding factors for the LPR diagnosis were excluded. While this may limit the generalizability of the study results to the whole population of patients with LPR, it also supports that the correct selection of LPR patients is essential to obtain a prompt treatment response, both subjectively and objectively.

Alginates act on LPR by means of three mechanisms: forming a raft floating over gastric contents that can be maintained within the stomach, creating a mechanical barrier that displaces the postprandial acid pocket, and binding pepsin and bile to potentially remove them from the refluxed material [31–38]. The formulations of different alginate suspensions tested in RCTs are compared in Table 2. Due to the differences in the formulation, results on the efficacy of one alginate suspension can not be generalized to other commercially available alginate suspensions. The study supports the non-inferiority of the *Gastrotuss®* to the PPIs in the improvement of LPR symptoms and signs. Nevertheless, along with efficacy, tolerability and adherence are essential outcomes for the use of a treatment in daily clinical practice. The alginate suspension was reported to be well tolerated by patients and only 2 mild adverse events were referred. Interestingly, the adherence of the *Gastrotuss®* was similar to the adherence of the PPI despite the higher number of daily doses (three daily doses vs one daily dose). Therefore, the comparable adherence and the good tolerability, further promotes the use of the alginates as an alternative to PPIs.

**Table 2:** Formulation of alginates tested in clinical trials

Alginate	Formulation
Gastrotuss	Magnesium Alginate, Simethicone, Fructose, Xanthan Gum, Honey, D-Panthenol, Fluid Extracts of Althea Officinalis and Papaver Rhoeas, Zinc Oxide, Sodium Bicarbonate, Sodium Hydroxide, Sodium Methyl p-Hydroxybenzoate, Sodium Propyl p-Hydroxybenzoate, Natural Flavourings, Erytrosine (E127), Purified Water
Gaviscon	Sodium alginate, sodium bicarbonate, methyl parahydroxybenzoate (E218), propyl parahydroxybenzoate (E216), calcium carbonate, carbomers, sodium saccharin, flavour fennel, sodium hydroxide, erythrosine, purified water
Alginos	Sodium alginates, Sodium bicarbonate, Calcium Carbonate, methylparaben, proprylparaben, sucralose, carbomer934P, sodium hydroxide, edetate disodium, essence of strawberry, purofied water

The study is not without limitations. Firstly, the originally calculated sample size was not achieved due to the spread of the SARS-COV-2. Thus, the non-inferiority margin was revised to achieve an adequate power with the recruited sample size. Therefore, the present results must be confirmed by larger samples. The diagnosis of LPR was empirical and lacked an impedance-pH metry. The advantages of the impedance-pH metry in the diagnosis of LPR are its objectivity and the possibility to identify different LPR subtypes. Nevertheless, this is not always practical and not well tolerated with a significant number of patients refusing to undergo impedance-pH metry, difficult to interpret, and, due to the intermittence of the disease, may lead to different results over time [39]. Thus, the combination of clinically relevant signs and symptoms and the exclusion of confounding concomitant disease should have ensured the correct selection of LPR patients and represents standard practice in most out-patient clinics. The study design was limited by the lack of a control group receiving only lifestyle recommendations. Additionally, the adherence of patients to these recommendations was not systematically recorded. Literature has demonstrated a significant improvement of LPR with behavioural changes [27]. Therefore, definite conclusions on whether the reduction of LPR signs and symptoms can be ascribed to the pharmacological treatments or to the adherence to lifestyle recommendations or to a combination of both treatments can not be drawn. Another limitation is the lack of patients’ blinding to the treatment. Because of the subjective nature of the primary study outcome, the significant change of the RSI may be the result of a placebo effect. However, the fact that the RFS, rated by a blinded clinician, also significantly improved seems to confirm the efficacy of the LPR treatments. The PPI dose was set at 20 mg of omeprazole per day, according to other studies comparing the effects of alginates to the effect of PPIs in gastroesophageal reflux [40–42]. Nevertheless, several other studies on LPR use higher doses of PPI [43]. Finally, the treatment period was short and there was no long-term follow-up. Future studies should expand the investigation to the longer treatment periods and compare the alginates to higher doses of PPI to verify if the non-inferiority of the alginates would be confirmed and the adherence and tolerability of both treatments would be comparable also in the long-term.

## Conclusion

The present RCT suggests that, after 2 months of treatment, LPR symptoms and signs are significantly reduced by both the PPI and the alginate suspension *Gastrotuss®*, when associated with diet and lifestyle modifications. The efficacy of *Gastrotuss®* was non-inferior to the efficacy of PPIs according to a margin of 7 points. Both treatments were well tolerated, and adherence was satisfactory. Thus, alginates seem to represent a valid alternative to PPIs for the treatment of LPR, potentially avoiding the adverse events associated to the long-term use of PPIs. Further studies with larger sample size, longer follow-up, and comparing the use of *Gastrotuss*^*®*^ to the placebo or to behavioural recommendations alone are warranted to confirm the present results.

## Data Availability

Data are available contacting the corresponding author upon reasonable request

## Appendix

Diet and lifestyle recommendations, according to the guidelines of the American College of Gastroenterology [22]

- Raise the head by 30 degrees during sleep
- Avoid laying down within 2 hours of eating a meal or drinking fluids
- Reduce body weight (in case of a BMI> 25 kg/m2 or in case of weight gain in the last few months)
